# The Prognosis for Delayed Immune Recovery in HIV-Infected Children might be Associated with Pre-cART CD4 + T cell Count Irrespective of Co-Infection with Tuberculosis

**DOI:** 10.21203/rs.3.rs-4243586/v1

**Published:** 2024-04-19

**Authors:** Funsho Ogunshola, Ruhul Khan, Musie Ghebremichael

**Affiliations:** Harvard University; University of Arizona; Harvard University

**Keywords:** HIV, TB, Immune recovery, CD4+ T cell count, cART Initiation, Piecewise Linear, Mixed-Effects Models

## Abstract

**Background:**

Immune reconstitution following the initiation of combination antiretroviral therapy (cART) significantly impacts the prognosis of individuals infected with human immunodeficiency virus (HIV). Our previous studies have indicated that the baseline CD4^+^ T cells count and percentage before cART initiation are predictors of immune recovery in TB-negative children infected with HIV, with TB co-infection potentially causing a delay in immune recovery. However, it remains unclear whether these predictors consistently impact immune reconstitution during long-term intensive cART treatment in TB-negative/positive children infected with HIV.

**Results:**

We confirmed that the baseline CD4^+^ T cell count is a significant predictor of immune recovery following long-term intensive cART treatment among children aged 5 to 18 years. Children with lower CD4^+^ T cell count prior cART initiation did not show substantial immunological recovery during the follow-up period. Interestingly, children who were co-infected with TB and had higher baseline CD4^+^ T cell count eventually achieved good immunological recovery comparable to the TB-negative HIV-infected children. Hence, the baseline CD4^+^ T cell count at the onset of treatment serves as a reliable predictor of immunological reconstitution in HIV-infected children with or without TB co-infection. Taken together, this follow-up study validates our previous findings and further establishes that initiating cART early alongside early HIV testing can help prevent the diminished CD4^+^ T cell count associated with inadequate immunological reconstitution.

## Introduction

Human immunodeficiency virus (HIV) targets the immune system and weakens defense against opportunistic infections such as Tuberculosis (TB). Individuals infected with HIV are not only at high risk of developing TB, but also at increased risk for severe forms of diseases as seen in the recent SARS-CoV-2 pandemic^1^. Despite intensive efforts for early diagnosis, prevention and treatment, HIV/TB co-infection remains a major public health concern in many parts of the world, particularly in the developing countries^2^. The use of combination antiretroviral therapy (cART) against HIV is one of the strategies recommended by the World Health Organization (WHO) to prevent TB^3,4^. This strategy has significantly transformed the deadly effects of HIV infection into a manageable disease by suppressing viral replication, promoting the recovery of CD4^+^ T cell count, and improving the survival and overall quality of life of HIV-infected individuals^5^. Although cART has demonstrated a significant reduction in the activation of infected cells among people living with HIV (PLHIV), an estimated 15–30% of PLHIV still struggle to attain optimal recovery of CD4^+^ T cell count despite undergoing intensive cART treatment^6,7^. In addition, the incidence of TB among cART-treated people remains considerably higher than in the general population, indicating an underlying immunological dysfunction^8^. While the risk factors for active TB have been linked to immunological dysfunction in HIV-infected people who have not received cART^5^, little is known for cART-treated individuals. For instance, it is unclear whether suboptimal virological suppression or poor immunological recovery following intensive cART treatment increases the residual risk of TB reactivation/acquisition. Additionally, while partial immune recovery contributes to the control of opportunistic infections, the clinical picture in the case of HIV/TB co-infection is different, as severe active TB infections can occur even at high CD4^+^ T cell count^9^. To date, there is no therapy that can effectively restore CD4^+^ T cell count to normal levels. Thus, further studies to characterize CD4^+^ T cell recovery among PLHIV with or without TB co-infection are urgently needed.

The hallmark of HIV infection is the depletion of CD4^+^ T cells, which is an important contributor to the increased risk of developing active TB^10^. HIV infection can result in changes across T cell subsets with altered differentiation profiles and increased levels of immune activation, which persist even in individuals on/after cART^11,12^. Continuous HIV stimulation and activation render T cells dysfunctional^13^, and the necessary diversification and balance of T cell populations, important to ensure maintenance of immune homeostasis, is progressively lost^14^. Among others, thymus production of CD4^+^ and new naive T cells, as well as proliferation of peripheral naive T cells become progressively exhausted/impaired as the infection progresses^15^, rendering the body immunocompromised and unable to combat opportunistic infections.

It is widely accepted that CD4^+^ T cell count following cART initiation is the most important predictor of immune recovery^5^. Adequate immune recovery is defined as the attainment of CD4^+^ T cell count within the range observed in healthy adult individuals (i.e., 500–1500 cell/ul)^16^. Our earlier work demonstrated that the baseline CD4^+^ T cell count and percentage are reliable predictors for infants attaining this status^17^. However, the dynamics of immune reconstitution of PLHIV under long-term cART seems to vary among different populations and regions^18^. Early studies indicate that CD4^+^ T cell recovery is sustained for more than 3 years in individuals with advanced HIV-1 infection who receive cART^5,19^. Within the first three to six months of intensive cART, there is typically a significant increase in CD4^+^ T cell count, which is followed by a second phase of slower increase in most cases^20^. Notably, these studies mostly focus on HIV infected individuals, and there is limited knowledge on the long-term impact of cART treatment on immune recovery in HIV/TB co-infected individuals. In general, the lower the CD4^+^ T baseline cell count are when cART is initiated, the longer it takes to reach desired levels of immune recovery^21,22^. Investigations have suggested that CD4^+^ T cell recovery may plateau before the physiological range is reached, especially in those who start cART at a very low CD4^+^ T cell count^22,23^. Determining the impact of early cART initiation on immunological recovery in individuals co-infected with TB will provide essential information for effective monitoring and treatment of this population.

Whereas cART treated individuals with suboptimal immune recovery are referred to as “discordant immune responders”^24^, the underlying mechanisms for this discrepant post-cART developments are still ill-defined, with discordant immune recovery being associated with a number of factors such as ageing, lower nadir CD4^+^ T cell count, residual viral replication, increase T cell death, immune hyperactivation, altered ratio of regulatory T cells to Th17 cells, tissue fibrosis and specific metabolic profiles^25–27^. TB co-infection has also been proposed as a potential factor that can affect the long-term chances of immune recovery through increased apoptosis of CD4^+^ T cells^1,28,29^. Thus, clinical events, in particular, the kinetics of CD4^+^ T cell recovery in cART treated PLHIV with or without TB will be highly informative in predicting immune recovery. In our previous analysis of baseline data from HIV-infected children, we reported that the baseline CD4^+^ T cell count could predict immune recovery^17^. In this follow-up study, we explored historical data both pre- and post treatment initiation to elucidate the dynamics of immune recovery among HIV-infected children with or without TB co-infection following extensive cART treatment.

## Materials and Methods

### Study participants

The study included data collected from a retrospective study of HIV-positive children. The children were between 0–13 years of age, received cART between June 2004 and December 2009 in Accra, Ghana. All the children were on their first-line regimen of nonnucleoside analog-based cART consisting of zidovudine (AZT) or stavudine (d4T) plus lamivudine (3TC), plus either nevirapine (NVP) or efavirenz (EFV). The children diagnosed with active TB at the time of cART treatment initiation received simultaneous treatment for TB alongside cART. Participant’s characteristics such as age and gender were collected at study entry. Moreover, CD4^+^ T-lymphocyte count and percentage were quantified after surface staining (CD3^+^ CD4^+^) by standard flow cytometry using a FACS Count system (Becton-Dickinson, Franklin Lakes, NJ) at Korle-Bu Teaching Hospital in samples collected before and after the initiation of cART. A patient was defined as having achieved immune recovery if they reached and maintained a target CD4^+^ T-lymphocyte percentage of 25% following the initiation of cART^30,31^. The rationale, organization, and recruitment of the subjects, procedures used for quantification of CD4^+^ T-lymphocyte have been described previously^30,32^. The study protocol was approved by the Institutional Review Board of the Yale University School of Medicine and the University of Ghana Medical School.

### Statistical Analysis

Descriptive measures (such as frequency, percent, median and IQR) were used to summarize data. Analysis of repeated measures, using piecewise linear mixed-effects models, was conducted to assess the overtime change in CD4^+^ T cell count and compare the change in CD4^+^ T cell count by immune recovery status, adjusting for potential confounders. TB status and age at study entry were included as time-independent covariates in the model. The model allowed for different rates of CD4^+^ T cell changes during the pre-treatment and post-treatment phases of the study. Moreover, the model allowed the intercept and the rate at which CD4^+^ T cell count changes over time to vary across participants. Further, the model does not require participants to have the same number of visits or measurements and uses all available data instead of eliminating subjects with missing data, resulting in unbiased estimates of the model parameters when data were missing at random.

## Results

The analysis included a total of two-hundred thirty-four (n = 234) HIV-infected children who initiated cART regimens and had at least one post-cART CD4^+^ T-lymphocyte count. These children were followed up during the study period until they achieved immune recovery or were censored on the last day of contact. Most study participants achieved immune recovery (n = 171, 73%) during the study period. However, 27% percent (n = 63) failed to achieve immune recovery. Fifty-two percent of the participants (n = 121) were TB-positive at study entry and 50% of them were males. There was no statistically significant difference in gender (p = 0.5570) and TB (p = 0.2381) rates between the recovered and non-recovered patients. The median age of participants at study entry was 5.71 years, (IQR: 2.77–7.82); the recovered patients were younger compared to the non-recovered patients. Median baseline CD4^+^ T-cell count were significantly higher (p = 0.0014) in patients who recovered (484 cells/mm^3^; IQR = 274–892 cells/mm^3^) compared to patients who did not achieve immune recovery (279 cells/mm^3^; IQR = 76–517 cells/mm^3^).

**Figure 1:**
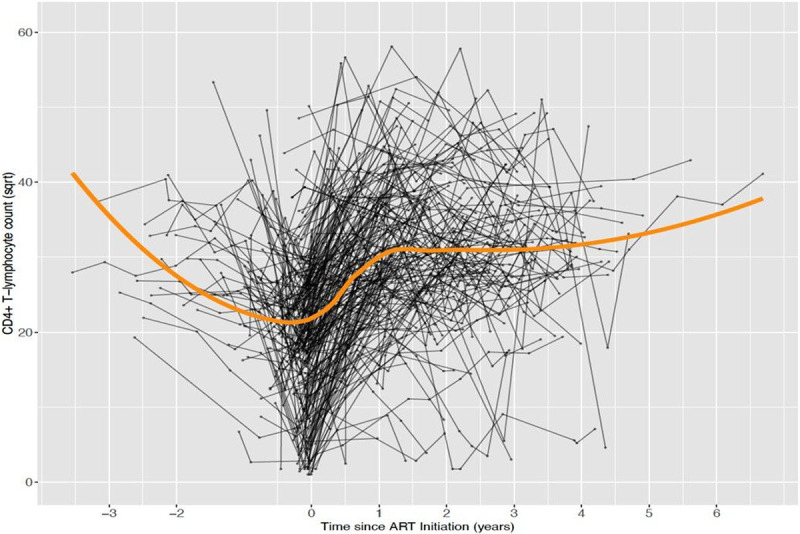
CD4+ T-cell count trajectories among children treated at Korle-Bu Teaching Hospital.

Figure 1 displays the pre-cART and post-cART longitudinal CD4^+^ T-cell count for the study participants. There was an overall decrease in pre-treatment CD4^+^ T-cell counts over time, with an average rate of decrease of 1.53 ± 0.75 cells/mm^3^ per year (p < 0.0420). However, post-treatment CD4^+^ T-cell count increased over time, with an increased average rate of 3.78 ± 0.29 cells/mm^3^ per year (p < 0.0001).

**Figure 2:**
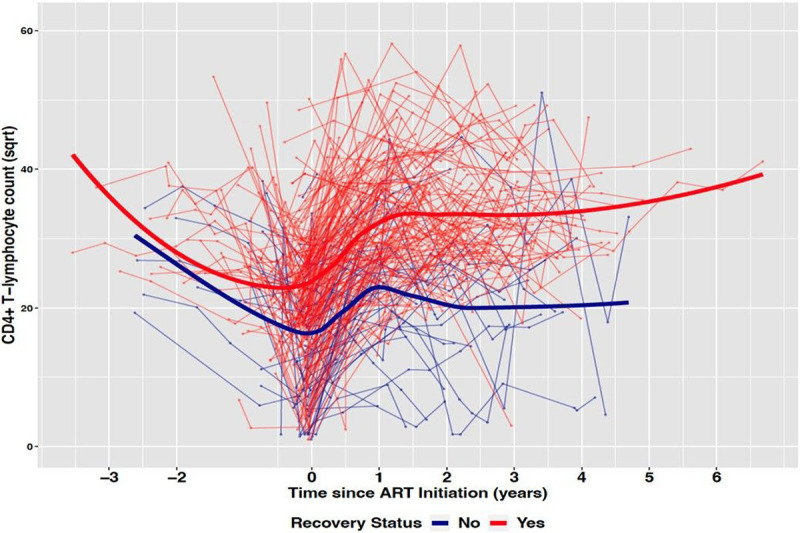
CD4+ T-cell count trajectories by recovery status among children treated at Korle-Bu Teaching Hospital.

Figure 2 presents the pre-cART and post-cART longitudinal CD4^+^ T-cell count for the study participants by immune recovery status. There was a significant difference (Δ) in CD4^+^ T-cell count at baseline between the two groups (Δ=7.67 cells/mm^3^, p < 0.0001). In both groups, there was a decline in CD4^+^ T-cell count before treatment initiation. The rate of pre-cART decline was significant for the non-recovered group (4.82 cells/mm^3^ per year; p = 0.0001), but not for the recovered group (0.24 cells/mm^3^ per year; p = 0.7742). The difference in pre-cART rates of CD4^+^ T-cell count decline was significantly different between the two groups (Δ=4.58 cells/mm^3^; p = 0.005). Although CD4^+^ T-cell count significantly increased in both groups post-cART treatment, the rate of increase was higher in the recovered group (3.75 cells/mm^3^ per year; p < 0.0001) compared to the rates in the non-recovered group (1.74 cells/mm^3^ per year; p = 0.003). The difference in these post-cART rates of CD4^+^ T-cell count increase was significantly different between the two groups (Δ=2.01 cells/mm^3^; p = 0.0021).

**Figure 3:**
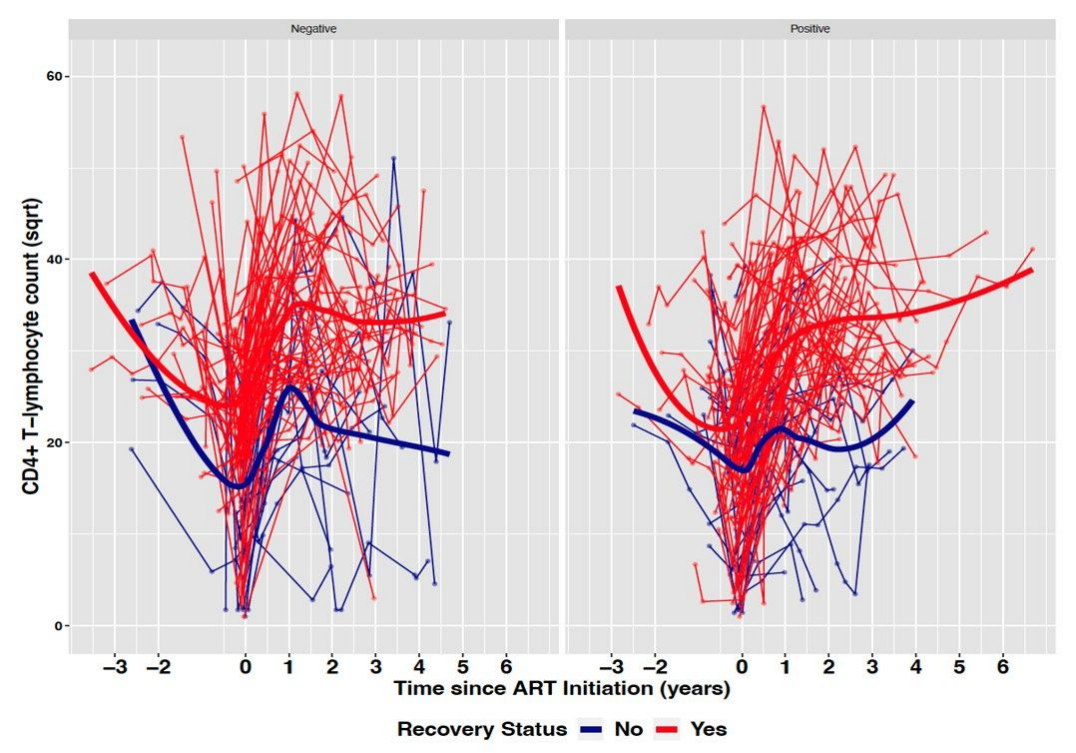
CD4+ T-cell counts trajectories by recovery status among TB negative (left) and TB positive (right) children treated at Korle-Bu Teaching Hospital.

In Fig. 3 the pre-cART and post-cART longitudinal CD4^+^ T-cell count of both groups were stratified based on the TB status. Overall, TB status did not alter the results observed in Fig. 2 thereby indicating that pre-cART CD4^+^ T-cell count is a significant determinant of immune recovery in our dataset. Notably, the increase in post-cART CD4^+^ T-cell count was not significant in the non-recovered group that are TB negative (0.96 cells/mm^3^ per year; p = 0.23).

## Discussion

HIV cART treatment has been proven to be effective in stabilizing and reconstituting CD4^+^ T cell levels and thus preventing progression to AIDS^33,34,35^. However, not all cART-treated individuals successfully experience immune reconstitution. In this follow-up study of our prior work^17^, we performed a detailed longitudinal analysis of CD4^+^ T cell count on historical data collected from children who were followed up for at least 4 years of intensive cART treatment. We used a rigorous statistical modelling analysis to define how intensive cART treatment shapes the kinetics of CD4^+^ T cell count in HIV-infected infants with or without active TB in order to determine the main parameters informing immune recovery, particularly in HIV/TB co-infected children. The high-endemicity of HIV/TB co-infections in sub-Saharan Africa complicates effective treatment of HIV, increases the risk of developing AIDS^36^, and altogether makes it difficult to accurately monitor the progression of these individuals following long-term intensive cART treatment, further highlighting the need for better clinical models of immune recovery that can be helpful in predicting patients’ outcomes, in particularly in resource-limited settings.

We analyzed the clinical parameters collected before and after intensive cART treatment for a cohort of 234 children, including the time of cART treatment initiation and their strict adherence to the treatment regimen. We found that regardless of the TB status, baseline CD4^+^ T cell count has the most significant impact on immune recovery.

We previously reported that CD4^+^ T cell count at the time of treatment initiation could predict immune recovery^17^, particularly in TB-negative individuals. Here, we describe baseline CD4^+^ T cell count as a biomarker for predicting immune recovery, confirming our previous findings while taking into account/exploring the longitudinal development of immunity over a prolonged amount of time. Notably, post-cART immune recovery was not different across HIV-infected individuals regardless of TB infection status. Although the TB-negative children had higher average baseline CD4^+^ T cell count, in line with our earlier findings^17^, we did not observe any statistically significant difference in the CD4^+^ T cell count trajectory between the two groups throughout the follow-up study period.

Participants with the highest baseline CD4^+^ T cell count, regardless of their TB status, had the best trajectory of CD4^+^ T cell count and recovered after long-term intensive treatment, consistent with the findings from earlier study^37^. Another study from sub-Saharan Africa has also reported no difference in the immune recovery of HIV-infected individuals with or without TB co-infection after starting cART^38^. These findings contrast with reports from high-income settings, which have linked the failure of immune recovery among cART-treated individuals to the levels of viral suppression^39,40^. These divergent results are probably affected by additional variables, such as the quality and accessibility of public health systems, genetic diversity, degrees of virological suppression, the timing of treatment initiation, and the specific type of cART provided to individuals across various cohort settings. Our study’s significant limitation will be addressed in future research that integrates multi-region sampling and longitudinal analysis among cART-treated individuals, with or without TB co-infection.

In this study, we rediscovered pre-cART CD4^+^ T cell count as an important biomarker that should be considered in predicting the fate of immune recovery following cART in infants, corroborating previous findings^17,32^. By predicting immune recovery, these findings can help improve the standard of care, particularly in resource-limited settings. A longitudinal investigation of CD4^+^ T cell phenotypes of HIV-infected persons with or without TB co-infection will help dissect the specific mechanisms leading to different recovery status in these individuals.

## Conclusions

An important process that happens in PLHIV after cART treatment is immune reconstitution, which is indicated by an increase in the CD4^+^ T cell count. Success of intensive cART can be translated as sustained recovery of CD4^+^ T cells, which is the primary surrogate marker used in clinical practice. We used longitudinal data collected from low-income settings to evaluate the trajectory of immune recovery and validate our earlier findings on using baseline CD4^+^ T cell count to predict immune recovery following cART. We followed 234 HIV-infected children with or without TB co-infection before and after the start of cART from the cohort we described in our previous study. Our findings show that pediatric children’s immune recovery is reliably predicted by baseline CD4^+^ T cell count. Our analysis revealed that children with lower CD4^+^ T cell count before treatment initiation did not experience immune recovery during the follow-up period, and the impact of TB co-infection on immunological recovery is minimal. Taken together, the findings further highlight the importance of offering/starting cART treatment as soon as patient is diagnosed with HIV which is pivotal to managing persons infected with HIV.

### Study limitation

It is important to acknowledge that this study is retrospective containing historical data we described in our previous study, and certain data were unavailable during the data analysis. Information such as TB treatment, and treatment history before the initiation of this study are all critical for making conclusive statements about the impact of TB on immune recovery. Therefore, findings from this study must be interpreted cautiously.

## Data Availability

The dataset used in the manuscript is available upon request from the corresponding author.

## Abbreviations

HIV: Human immunodeficiency virus
PLHIV: People living with HIV
AIDS: Acquired Immunodeficiency Syndrome
TB: Tuberculosis
cART: Combination Antiretroviral Therapy
AZT: Zidovudine
d4T: Stavudine
3TC: lamivudine
NVP: nevirapine
EFV: efavirenz
FACS: Fluorescence-activated cell sorting
IQR: Interquartile range
